# Boy Labour in Mines

**Published:** 1920-05-01

**Authors:** 


### THE PUBLIC WELL-BEING.

### BOY LABOUR IN MINES.

### Effect on Physical Development.

One rarely pauses to consider what lies behind the smooth surface of daily routine. Mr. Masefield tells in "Dauber" of the agony that the seaman had to undergo, in the days of the old wind-jammer, in order that trade might flow evenly for unadventurous folk; and something unguessed makes it dangerous for an introspective person to think too closely about those who feed and clothe and warm him. In the snug security of a fire-bright room it is disturbing to think of the men and boys who work in the darkness and danger of the mines, where gas and dust and "stone" hold hourly potentialities of disaster. But now and then these things must be faced; and in facing them one has at any rate the consolation of knowing that conditions steadily improve, and that many a boy who to-day begins his work in the mine at the age of fourteen has a father who began at eight or nine and worked longer hours in conditions more difficult and dangerous.

In different coal-fields the circumstances of work differ, but in no mine does the "half-time" system prevail—the system which allows children to spend half their time in school and half at factory work. And no boy may begin work in a mine before the age of fourteen except in a few cases where a certificate is granted to show that a certain educational stage has been passed. In Germany before the war no boy was allowed underground till the age of sixteen had been reached. Most mines are worked on the system of three shifts a day, but boy workers are not allowed on the night shifts. The working day is seven hours.

There is no rule which regulates the work a boy shall do on first entering the mine. Custom varies widely. In some districts a boy is not allowed to work at the coal face until he reaches the age of eighteen—the age at which, under the Sankey award, the full advance of wages was given. In other districts a boy works at the coal face straight away. Many of those best qualified to know, consider that even at eighteen years of age the strain of full mining work is too heavy and retards or definitely limits a youth's development. Many boys, of course, take up comparatively light work on first entering a mine; they look after the doors which regulate ventilation; and later become pony boys, taking charge of the animals which draw the trains of tubs filled by the miners. Another job is looking after the sidings of "landings" where the empty tubs are assembled, and despatching them to the various working places. In other mines boys do the "hauling," as it is still called, though the actual action is pushing. The tubs which are moved by this means contain anything from five or six up to ten cwt. Obviously, the economic limit up to which it would be profitable to push trucks manually is soon reached, and for distances more than about sixty yards other means of haulage are employed.

#### A Trying Job.

One of the most disagreeable and not the least dangerous of the jobs a pit boy is called upon to perform is known in some districts as "putting." "Putting" means taking the tubs from the road into the freshly-dug working places in the coal-face. The way in is often so narrow and so low that a pony is unable to enter; the workers have to crawl in one by one, and often the tub sticks fast against the roof or sides. The boy has to take in the empty tubs and take out the full ones. It may well be imagined that manipulating a tub loaded with half a ton of coal in circumstances of this sort is no light work for a boy; and it is work that has its dangers, too, when the roof is very low and the "stone" loose.

By and by the pit boy, who did not actually begin his mining career at the coal face, takes to doing a little shovelling, and, using his eyes, learns the knacks of his elders. In the matter of wages custom varies. A boy, on beginning work in a mine, is usually able to count on about one-third of a man's wages, and progress after that depends largely on district custom. In cases where the boy begins at once on the coal-face it is not unusual for him to be earning a man's wages within a year or two.

Although so much has been done to make conditions in the mines more healthy for juvenile as for adult labour, the same question arises as in the case of half-time factory workers—Should boys be allowed to do this work in any circumstances? Improved machinery would doubtless make a great deal of boy labour unnecessary. Mechanical haulage would do away at once with pit ponies and with pony boys; and in other directions machines could be saving the precious years which the boys might spend at school.

